# How accurate is anatomic limb alignment in predicting mechanical limb alignment after total knee arthroplasty?

**DOI:** 10.1186/s12891-015-0756-2

**Published:** 2015-10-27

**Authors:** Seung Ah Lee, Sang-Hee Choi, Moon Jong Chang

**Affiliations:** Department of Physical Medicine and Rehabilitation, College of Medicine, Kyung Hee University, Seoul, Republic of Korea; Department of Radiology, Samsung Medical Center, Sungkyunkwan University School of Medicine, Seoul, Republic of Korea; Joint Reconstruction Center, Gwangmyeong Saeum Hospital, Gyeonggi-do, Republic of Korea

**Keywords:** Anatomic alignment, Mechanical alignment, Accuracy, Outcome, Total knee arthroplasty

## Abstract

**Background:**

Anatomic limb alignment often differs from mechanical limb alignment after total knee arthroplasty (TKA). We sought to assess the accuracy, specificity, and sensitivity for each of three commonly used ranges for anatomic limb alignment (3-9°, 5-10° and 2-10°) in predicting an acceptable range (neutral ± 3°) for mechanical limb alignment after TKA. We also assessed whether the accuracy of anatomic limb alignment was affected by anatomic variation.

**Methods:**

This retrospective study included 314 primary TKAs. The alignment of the limb was measured with both anatomic and mechanical methods of measurement. We also measured anatomic variation, including the femoral bowing angle, tibial bowing angle, and neck-shaft angle of the femur. All angles were measured on the same full-length standing anteroposterior radiographs. The accuracy, specificity, and sensitivity for each range of anatomic limb alignment were calculated and compared using mechanical limb alignment as the reference standard. The associations between the accuracy of anatomic limb alignment and anatomic variation were also determined.

**Results:**

The range of 2-10° for anatomic limb alignment showed the highest accuracy, but it was only 73 % (3-9°, 65 %; 5-10°, 67 %). The specificity of the 2-10° range was 81 %, which was higher than that of the other ranges (3-9°, 69 %; 5-10°, 67 %). However, the sensitivity of the 2-10° range to predict varus malalignment was only 16 % (3-9°, 35 %; 5-10°, 68 %). In addition, the sensitivity of the 2-10° range to predict valgus malalignment was only 43 % (3-9°, 71 %; 5-10°, 43 %). The accuracy of anatomical limb alignment was lower for knees with greater femoral (odds ratio = 1.2) and tibial (odds ratio = 1.2) bowing.

**Conclusions:**

Anatomic limb alignment did not accurately predict mechanical limb alignment after TKA, and its accuracy was affected by anatomic variation. Thus, alignment after TKA should be assessed by measuring mechanical alignment rather than anatomic alignment.

## Background

Coronal alignment of the lower limb is a major determinants of successful total knee arthroplasty (TKA) [1–3], and mechanical limb alignment is considered the gold standard in the assessment of coronal alignment after TKA [4–7]. Many recent studies have used mechanical limb alignment to assess radiographic outcomes after TKA. However, the measurement of mechanical limb alignment requires special equipment to check the full-length standing anteroposterior (AP) radiographs. In contrast, anatomic limb alignment can be measured on standard (14 × 17 inch) knee radiographs, which are readily available in most clinics. Thus, a number of large, multicenter studies with long-term follow-up periods have used anatomic limb alignment to assess radiographic outcomes [8–10]. However, anatomic limb alignment often differs from mechanical limb alignment, which can make it difficult to compare radiographic outcomes between studies that used different methods of measurement.

The alignment of the limb after TKA is often assessed by using an acceptable range for neutral alignment and using categorical analyses to determine the radiographic outcome. Previous studies have found that knees within an acceptable range for mechanical limb alignment (neutral ± 3°) show better clinical outcomes after TKA than knees for which the coronal alignment was out of this range [3]. Despite recent disagreement regarding the usefulness of this range [11, 12], mechanical limb alignment within ±3° of neutral is most frequently used as an acceptable range to assess the alignment of the lower limb after TKA. In contrast, there is no representative acceptable range for anatomic limb alignment. Given the physiological difference of 6° between the mechanical and anatomic axes of the femur, 6 ± 3° (i.e. 3–9°) may be reasonable [13, 14]. In contrast, the Knee Society Score (KSS) uses 5–10° as the acceptable range for anatomic limb alignment [15]. In addition, the new KSS, which has recently been devised, adopted 2–10° as the acceptable range for anatomic limb alignment [16, 17]. Nonetheless, there is a lack of information regarding which range for anatomic limb alignment can best predict the acceptable range for neutral mechanical limb alignment (neutral ± 3°) with the highest accuracy, specificity, and sensitivity.

The difference in alignment assessment between anatomic and mechanical alignments may be caused by deformities of the femur and/or the tibia [18]. For the femur, mechanical alignment is determined by measuring the line joining the center of the femoral head and the center of the femoral notch. Thus, mechanical alignment is not affected by anatomic variation [19]. In contrast, anatomic alignment uses the line bisecting the distal shaft of the femur. Thus, the degree of femoral bowing can influence the difference between the two methods. Tibia bowing can also affect the accuracy with which anatomic alignment predicts mechanical alignment [18]. Furthermore, the differences between the two types of alignment can be exaggerated by varus orientation of the femoral neck because the center of the femoral head is more medially located in varus deformity of the femoral neck.

We sought to assess the accuracy, specificity, and sensitivity of each of the three commonly used ranges for anatomic limb alignment (3–9°, 5–10° and 2–10°) in predicting an acceptable range (neutral ± 3°) for mechanical limb alignment after TKA. We also investigated whether the accuracy of anatomic limb alignment was affected by anatomic variation, such as the degree of femoral bowing, tibial bowing, and varus orientation of the femoral neck. We hypothesized that anatomic limb alignment would not accurately predict mechanical limb alignment for most knees and that the acceptable range for anatomic limb alignment in the new KSS (i.e., 2–10°) would show the highest accuracy. We also hypothesized that anatomic limb alignment would be less accurate in knees that had greater femoral bowing, tibial bowing, or varus orientation of the femoral neck.

## Methods

This retrospective study included 314 primary TKAs. From January to July 2011, 284 primary TKAs were performed at our institution. Because the vast majority of TKA candidates in Korea are women, we extended the review of medical records to include 87 knees from men who underwent primary TKA from August 2011 to December 2012. Thus, in total, 371 knees were considered for inclusion in this study. Of these, 57 knees were excluded for the following reasons: 1) 50 (13 %) knees had poor image quality in terms of rotation of the limb, 2) 4 (1 %) knees did not have full-length standing AP radiographs, and 3) 3 (1 %) knees underwent revision surgery within 1 year of the primary TKA. No patient had flexion contracture greater than 20° at 1-year follow-up. Finally, 314 primary TKAs in 212 patients were included in this study. Most knees (312; 99 %) had TKA due to osteoarthritis. The remaining 2 knees had TKA due to rheumatoid arthritis. There were 204 bilateral TKAs (65 %; 102 patients) and 110 unilateral TKAs (35 %; 110 patients). There were 150 (71 %) women and 62 (29 %) men with a mean age of 68 years (range, 52 to 84 years). The mean weight was 65 kg (range, 46 to 95 kg), and the mean height was 157 cm (range, 140 to 178 cm). The mean body mass index (BMI) was 26.6 kg/m^2^ (range, 18.4 to 39 kg/m^2^). The patients and/or their families were informed that data from the case would be submitted for publication, and gave their consent. All participants gave their informed consent to assessing and using their data. The study protocols were approved by the ethics committee of the Samsung Medical Center.

**Fig. 1:**
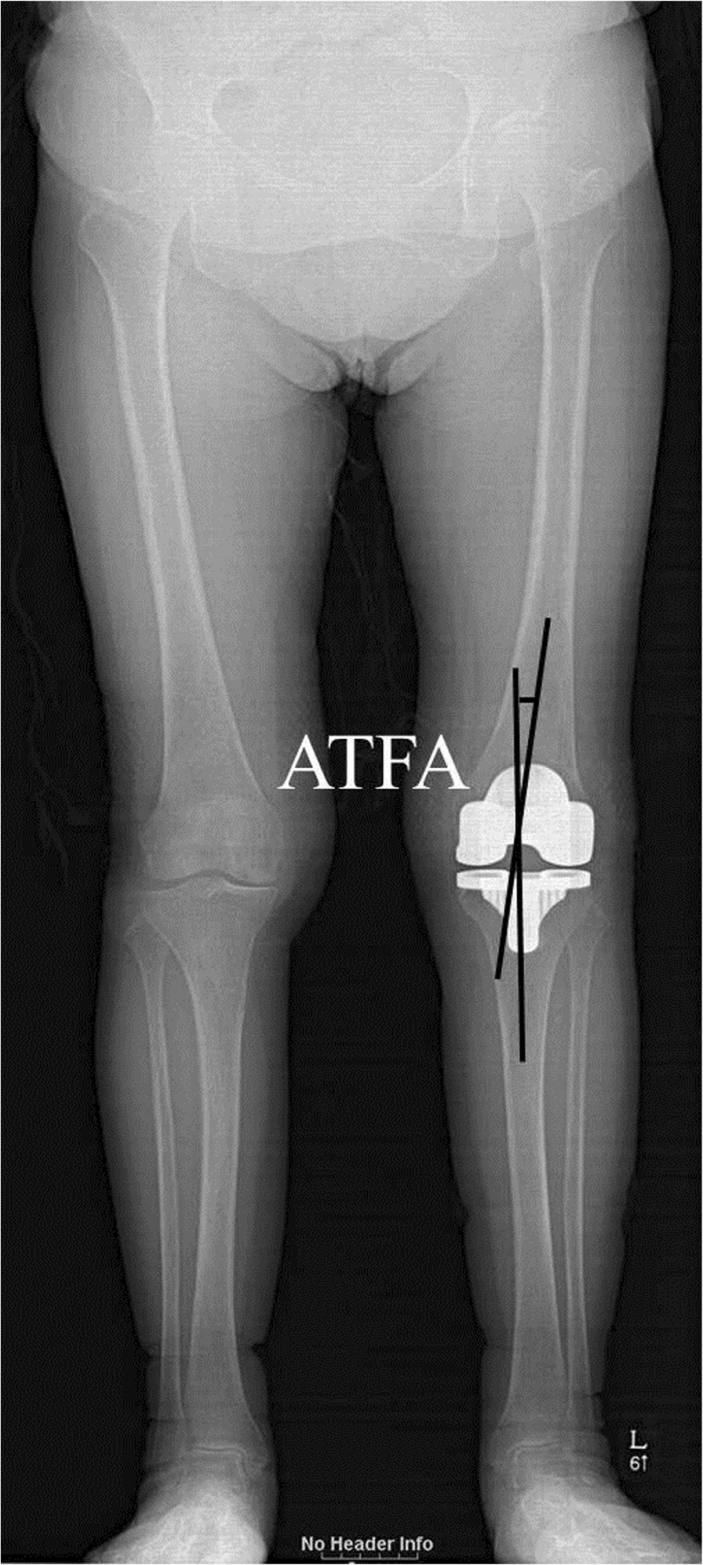
The anatomic tibiofemoral angle (ATFA) was measured as a surrogate of anatomic limb alignment. The anatomic femoral axis was identified by drawing a line between the notch center of the femoral components and a point 15 cm above the lowest point of the lateral femoral condyle, in the middle of the femoral shaft. The anatomic tibial axis was defined as a line joining the point on the bisector of the tibia, 15 cm below the highest point of the lateral tibial plateau and the center of the tibial component surface.

**Fig. 2:**
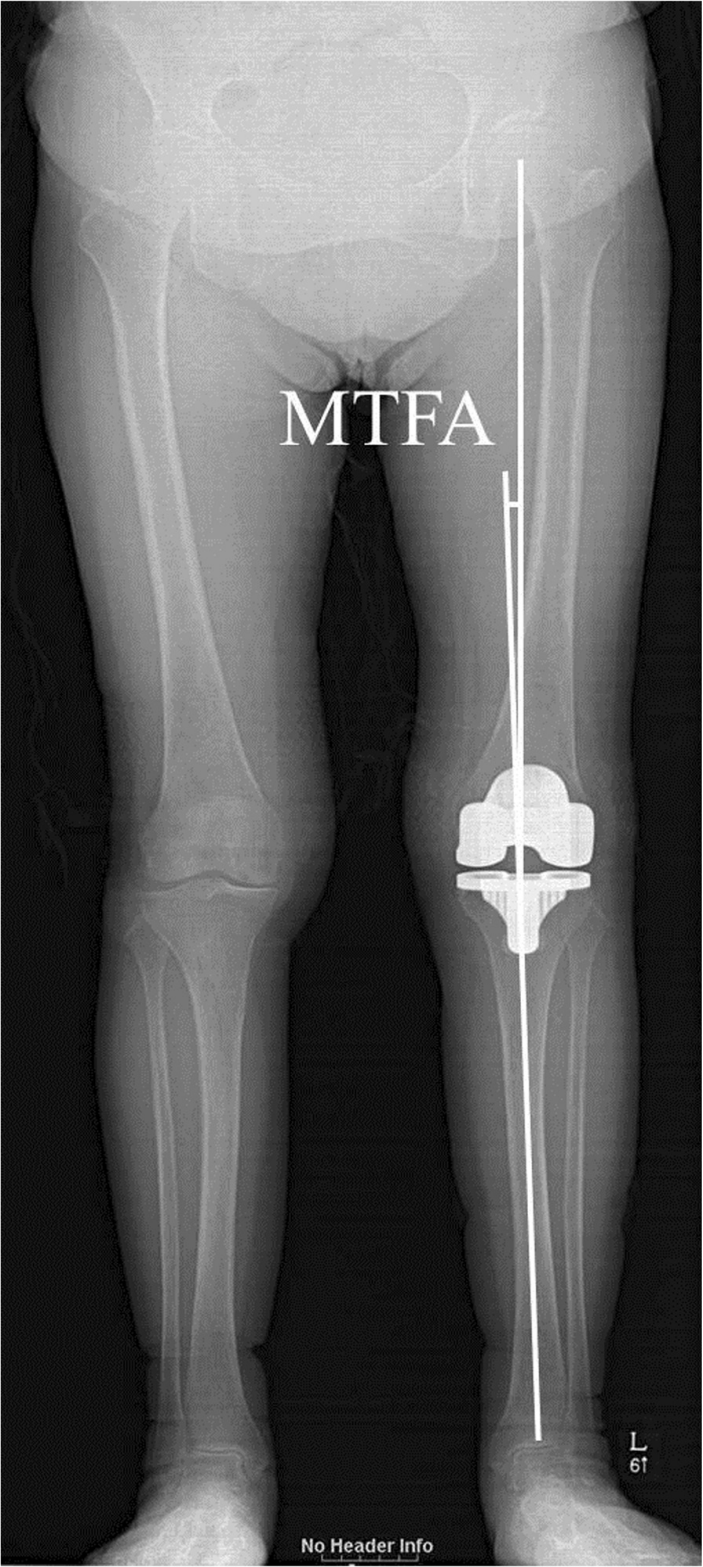
The mechanical tibiofemoral angle (MTFA) was defined as the angle formed between the mechanical axis of the femur and that of the tibia. The mechanical axis of the femur was defined as the line joining the center of the femoral head and the center of the femoral component. The mechanical axis of the tibia was defined as the line connecting the center of the tibial component and the center of the tibial plafond.

We measured the anatomic and mechanical limb alignments using the methods reported previously [4, 20, 21]. The anatomic tibiofemoral angle was defined as the angle formed between the anatomic femoral and tibial axes (Fig. 1). The anatomic femoral axis was identified by drawing a line between the notch center of the femoral components and a point 15 cm above the lowest point of the lateral femoral condyle, in the middle of the femoral shaft. The anatomic tibial axis was defined as the line joining the point on the bisector of the tibia, 15 cm below the highest point of the lateral tibial plateau and the center of the tibial component surface. The mechanical tibiofemoral angle was defined as the angle formed between the mechanical axis of the femur and that of the tibia (Fig. 2). The mechanical axis of the femur was defined as the line joining the center of the femoral head and the center of the femoral component. The mechanical axis of the tibia was defined as the line connecting the center of the tibial component and the center of the tibial plafond.

**Fig. 3:**
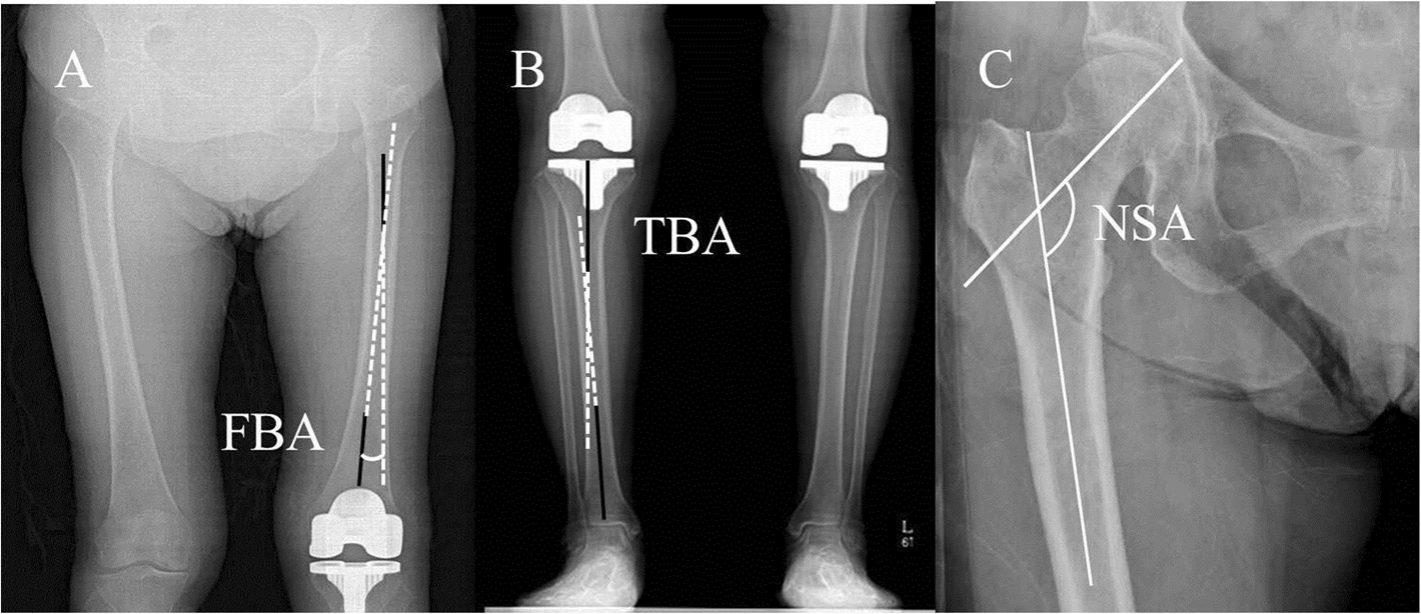
The femoral bowing angle (FBA) was defined as the angle made by the mid-diaphyseal lines of the proximal and distal parts of the femur (**a**). The tibial bowing angle (TBA) was defined as the angle made by the lines bisecting the proximal and distal parts of the tibia (**b**). The neck-shaft angle (NSA) of the femur was defined as the angle formed between the line bisecting the femoral neck and a bisector of the proximal diaphysis (**c**).

We also measured the femoral bowing angle, tibial bowing angle, and neck-shaft angle of the femur. The femoral bowing angle was defined as the angle made by the mid-diaphyseal lines of the proximal and distal portions of the femur [4, 20]. The line of the proximal femur was the line connecting the points at 0 and 5 cm below the lower end of the lesser trochanter. The line of the distal femur was the line connecting the points at 5 cm and 10 cm from the lowest portion of the lateral femoral condyle (Fig. 3a). In addition, the tibial bowing angle was defined as the angle made by the lines bisecting the proximal and distal portions of the tibia [19]. This angle was set to reflect the proximal tibia vara. The proximal line was defined as the line connecting the center of the tibial component and the point bisecting the tibia at 15 cm distal from the tibial component surface. The distal line was defined as the line connecting the tibial plafond center and the point 15 cm proximal from the plafond (Fig. 3b). The neck-shaft angle of the femur was defined as the angle formed between the line bisecting the femoral neck and a bisector of the proximal diaphysis (the line connecting the points at 0 and 15 cm distal from the piriformis fossa of the femur) (Fig. 3c). Negative values for anatomic and mechanical limb axes represented varus deformity. For femoral and tibial bowing, positive values represented lateral bowing (Table 1).

**Table 1:** Radiographic parameters

Parameter	Mean	SD	Minimum	Maximum
Mechanical tibiofemoral angle (°)	-1.0	2.4	-10.0	6.9
Anatomic tibiofemoral angle (°)	7.1	3.0	0.5	17.6
Femoral bowing angle (°)	-4.7	3.4	-15.3	0.1
Tibial bowing angle (°)	-4.1	2.8	-12.5	0.2
Neck-shaft angle (°)	124.1	4.4	111.6	139.5

*Abbreviation*: *SD* standard deviation

Both anatomic and mechanical alignments of the lower limb were measured on the same full-length standing AP radiographs taken 1 year after surgery. When the radiographs were checked, a reference template on the platform of radiography machine was used to control limb rotation, and the patient was asked to stand with the feet shoulder length apart. Radiographic measurements were performed with a picture archiving and communication system (PACS) (General Electric Medical systems, Milwaukee, WI). Alignment was measured to the nearest 0.1 mm for length measurements and 0.1° for angular measurements. The intra- and interobserver reliabilities of all measurements were determined by selecting 20 knees and measuring all angles twice (two weeks apart) by two observers (two of the authors). The reliability of the measurements was assessed with intraclass correlation coefficients (ICC). The ICCs for the intra- and interobserver reliability of the measurements were almost perfect (>0.9).

Statistical analyses were performed with SAS version 9.3 (SAS Institute, Cary, NC). For mechanical limb alignment, neutral ± 3° was considered to be the acceptable range. Applying this range to the knees in the present study revealed that mechanical limb alignment was within the acceptable range for 270 (86 %) knees. For anatomic limb alignment, three acceptable ranges were considered for this study: 3–9°, 5–10° and 2–10°. The accuracy of each range was calculated and compared with the angles for mechanical limb alignment, which were used as a reference standard. To determine the specificity and sensitivity for each range of anatomic limb alignment, varus or valgus malalignment using mechanical limb alignment were set as positive findings. In contrast, knees within an acceptable range of mechanical limb alignment were set as negative findings. Then, we calculated the specificity of each range of anatomic limb alignment. In addition, the sensitivity for varus or valgus malalignment was calculated separately. The results are presented as percentages and 95 % confidence intervals (CI). Statistical significance was determined with McNemar tests with Bonferroni corrections. To determine the associations between the accuracy of anatomic limb alignment and femoral bowing, tibial bowing, or varus orientation of the neck of the femur, only the 2–10° range was used as a dependent variable because it is the most recently recommended range of the Knee Society. Statistical significance was determined with multiple logistic regression analyses, and the results are presented as odds ratios (OR) and 95 % CI.

## Results

Although the 2-10° range for anatomic limb alignment showed the highest accuracy, anatomic alignment was not accurate in most knees with any of the three methods (3–9°, 65 %; 5–10°, 67 %; 2–10°, 73 %) (Table 2). The specificity of the 2–10° range was 81 %, and it was significantly higher than that of the other ranges (3–9°, 69 %; 5–10°, 67 %) (Table 3). However, the sensitivity of the 2–10° range to predict varus malalignment was only 16 %, and it was significantly lower than that of the other ranges (3–9°, 35 %; 5–10°, 68 %) (Table 4). In addition, the sensitivity of the 2–10° range to predict valgus malalignment was only 43 %, which was higher than its sensitivity for varus malalignment (Table 5).

**Table 2:** Accuracy of three commonly used ranges for anatomic limb alignment to predict the acceptable range (neutral ± 3°) for mechanical limb alignment

Parameter	Accuracy (%)	95 % CI	p-value	
			5-10°	2-10°
3-9°	65	59.5-70.0	1.000	<0.001
5-10°	67	61.2-71.6	NA	0.040
2-10°	73	67.4-77.3	NA	NA

*Abbreviations*: *CI* confidence interval; *NA* not applicable

**Table 3:** Specificity of three commonly used ranges for anatomic limb alignment to predict the acceptable range (neutral ± 3°) for mechanical limb alignment*

Parameter	Specificity (%)	95 % CI	P-value		Errors^†^	
			5-10°	2-10°	Varus	Valgus
3-9°	69	63.1-74.1	1.000	<0.001	11 (4)	73 (27)
5-10°	67	60.8-72.0	NA	<0.001	44 (16)	46 (17)
2-10°	81	76.0-85.3	NA	NA	5 (2)	46 (17)

*Mechanical limb alignment of 270 of 314 (86 %) knees was within neutral ± 3°. ^†^Data are presented as counts with proportions in parentheses; the knees were categorized into varus or valgus malalignment using the anatomic limb alignment method even if they were within the acceptable range using the mechanical limb alignment method

*Abbreviations*: *CI* confidence interval; *NA* not applicable

**Table 4:** Sensitivity of three commonly used ranges for anatomic limb alignment in predicting knees with varus malalignment using the mechanical limb alignment method*

Parameter	Sensitivity (%)	95 % CI	p-value	
			5-10°	2-10°
3-9°	35	21.8-51.2	<0.001	<0.001
5-10°	68	51.5-80.4	NA	<0.001
2-10°	16	7.6-31.1	NA	NA

*Mechanical limb alignment of 37 of 314 (12 %) knees showed varus malalignment

*Abbreviations*: *CI* confidence interval; *NA* not applicable

**Table 5:** Sensitivity of three commonly used ranges for anatomic limb alignment in predicting knees with valgus malalignment using the mechanical limb alignment method*

Parameter	Sensitivity (%)	95 % CI	p-value	
			5-10°	2-10°
3-9°	71	35.8-91.8	<0.001	<0.001
5-10°	43	15.8-75.0	NA	NA
2-10°	43	15.8-75.0	NA	NA

*Mechanical limb alignments of 7 of 314 (2 %) knees showed valgus malalignment

*Abbreviations*: *CI* confidence interval; *NA* not applicable

The accuracy of anatomic limb alignment was affected by the degree of femoral and tibial bowing, but not by the degree of varus orientation of the femoral neck. The accuracy of anatomic limb alignment was reduced in knees with greater femoral bowing (*p* < 0.001, OR 1.2, 95 % CI [1.1, 1.3]) and tibial bowing (*p* < 0.001, OR 1.2, 95 % CI [1.1, 1.3]). For each 1° increase in femoral or tibial bowing, the odds of inaccuracy were 1.2 times greater.

## Discussion

Coronal alignment of the lower limb is an important radiographic outcome variable after TKA [1, 3]. To determine coronal limb alignment, both anatomic limb alignment and mechanical alignment have been used. However, these two alignments often differ. Furthermore, no consensus exists regarding the acceptable range for anatomic limb alignment for the prediction of an acceptable range of mechanical limb alignment (neutral ± 3°). If anatomic alignment cannot accurately predict mechanical alignment, the results of clinical studies that use anatomical alignment are likely to be inaccurate. Thus, we sought to assess the accuracy, specificity and sensitivity of each of three commonly used ranges for anatomic limb alignment (3–9°, 5–10° and 2–10°) in predicting an acceptable range (neutral ± 3°) of mechanical limb alignment after TKA. We also aimed to determine whether the accuracy of anatomic limb alignment was affected by anatomic variation.

This study has several limitations. First, we only included patients from one Asian country. Thus, this study cannot provide information on the accuracy of anatomic alignment after TKA for other ethnicities. A previous study found that the relative difference between anatomic and mechanical alignment depends on the study population [4]. Furthermore, Asian patients are more likely to have femoral or tibial bowing than are Caucasians [4, 14]. Thus, the accuracy of anatomic limb alignment can differ according to ethnicity. However, considering that an increasing number of TKAs are being performed in Asian countries, we believe the present study provides valuable information to a broad readership. Second, 71 % of the subjects included in this study were women. The characteristics of bone geometry can differ between the sexes, and thus, caution should be used when applying our results to other populations with different sex ratios. However, we did attempt to enroll more men despite the extreme predominance of female TKA patients in our country [4, 22]. Third, this study only included radiographic results without clinical data. Thus, we do not know how the differences between the two methods affect clinical outcomes. We focused on determining the degree of difference and its characteristics between the radiographic data measured with the anatomic and mechanical limb alignments. In addtion, this study used two-dimensional assessment with conventional radiographs even though femoral and/or tibial bowing may also be affected by sagittal bowing of the bones and rational shapes. Similarly, flexion contracture of the knee joint can also affect the results of the two-dimensional study. Finally, our results may have been different if we had used a different range of anatomic limb alignments. However, we assessed the ranges proposed in the KSS, both the new and the old, which are the most popular scoring system in TKA [15, 17], so we believe that we chose the most appropriate ranges for our analyses.

Our findings support the hypothesis that anatomic limb alignment does not accurately predict mechanical limb alignment in most knees. Some previous studies have assessed the correlation between anatomic and mechanical limb alignment. These studies found moderate to excellent correlations (r = 0.65 to 0.86) and thus proposed that anatomic limb alignment can be used as a proxy for mechanical alignment [13, 23, 24]. However, the offset angles between anatomic and mechanical limb alignments were reported to have large variations (0.1 to 4.21°) in previous studies [5, 23–25]. In addition, the offset angles differed according to sex [4]. Therefore, even if moderate to excellent correlations exist between anatomic and mechanical limb alignments, the absolute values can differ considerably between the two methods. The inaccuracy of the anatomic alignment measurements was probably caused by the mismatch between the acceptable ranges for the two methods. Surgeons typically use femoral bushings with 5–6° of valgus during TKA on the assumption that the distal femoral mechanical-anatomical angles are 5–6°. On the basis of this assumption, an angle of 6 ± 3° is a reasonable range for acceptable anatomic limb alignment [13, 14]. However, a significant number of patients (28.6 %) have distal femoral mechanical-anatomical angles that are outside of the range of 5 ± 2° (range, 2.0 - 9.6°) [26]. Furthermore, the acceptable ranges for anatomic limb alignment used in previous studies have shown large variability [15–17, 20]. The desired anatomic limb alignment is defined as 2–10° in the new KSS score [16, 17]. Compared to the range of 5–10° in the old KSS [15], the range of 2–10° had substantially higher accuracy and specificity in the current study.

**Fig. 4:**
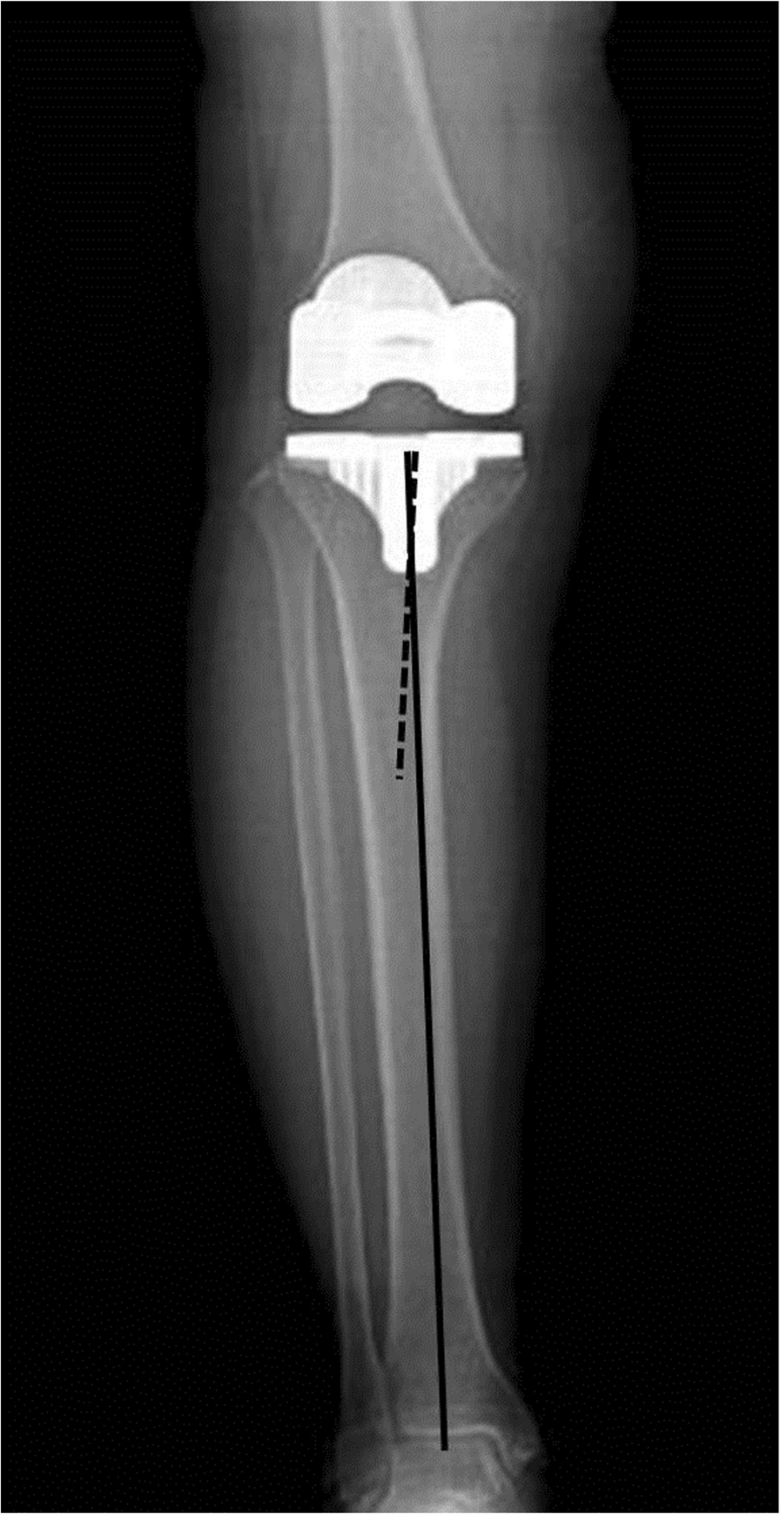
The anatomic alignment errors of the tibial components were probably caused by medialization of the proximal tibia relative to the distal shaft of the tibia due to the deformity of the proximal tibia vara. In this particular case, coronal alignment of the tibial component was interpreted as valgus malalignment using the anatomic alignment method even when the mechanical component alignment was within neutral ± 3.

The findings of this study affirm the hypothesis that anatomic limb alignment leads to lower accuracy in knees with greater femoral bowing or tibial bowing. Previous studies have found that femoral bowing is the anatomical characteristic that has the greatest effect on the difference between anatomic and mechanical limb alignments measurements [4, 13, 18, 19]. This finding is in agreement with ours. In addition, we found that tibial bowing led to a similar reduction in accuracy. If severe tibial bowing was present, the tibial axis was often measured as valgus malalignment when using the anatomic alignment method, even if the knee had an acceptable range of axis deviation with the mechanical alignment method (Table 3). This was probably caused by medialization of the proximal tibia relative to the distal shaft of the tibia because of the deformity of the proximal tibia vara (Fig. 4). Thus, our findings indicate that femoral and tibial bowing should be considered when evaluating limb alignment after TKA with the method of anatomic alignment.

## Conclusions

Anatomic limb alignment did not accurately predict mechanical limb alignment after TKA, and its accuracy was affected by anatomic variation. Thus, alignment after TKA should be assessed by measuring mechanical alignment rather than anatomic alignment. In addition, our findings should be considered when interpreting radiographic results on alignment of the limb after TKA.
